# Dual Roles and Therapeutic Prospects of Proximal Tubular Epithelial Cell Senescence in Acute Kidney Injury

**DOI:** 10.3390/biom16040611

**Published:** 2026-04-20

**Authors:** Yifan Qiao, Jin Zhao, Minna Liu, Jie Liu, Qiao Zheng, Ruotong Xu, Xiaoxuan Ning, Shiren Sun, Xiangmei Chen

**Affiliations:** 1 Department of Nephrology, Xijing Hospital, Fourth Military Medical University, No. 127 Changle West Road, Xi’an 710032, China; 2906943074@fmmu.edu.cn (Y.Q.); zhj_special@163.com (J.Z.); yadxlj12138@163.com (J.L.); z18892162629@163.com (Q.Z.); xrt136691@fmmu.edu.cn (R.X.); 2 Shaanxi Provincial Clinical Research Center for Kidney Diseases, Xi’an 710004, China; 3 Fundamental Medical Science Research Laboratories, the 940th Hospital Joint Logistics Support Forces of the People’s Liberation Army, Lanzhou 730050, China; lmn2010@foxmail.com; 4 Department of Geriatric, Xijing Hospital, Fourth Military Medical University, Xi’an710032, China; ningxx01@fmmu.edu.cn; 5 State Key Laboratory of Kidney Disease, Department of Nephrology, Chinese People’s Liberation Army General Hospital and Military Medical Postgraduate College, 28th Fuxing Road, Beijing 100853, China

**Keywords:** acute kidney injury, proximal tubular epithelial cells, acute cellular senescence, chronic cellular senescence, therapy

## Abstract

Acute kidney injury (AKI), a life-threatening disorder marked by abrupt renal dysfunction, is increasingly recognized as a global healthcare challenge. It not only triggers immediate organ dysfunction but also heightens long-term risks of chronic kidney disease (CKD). The senescence of proximal tubular epithelial cells (PTECs) has a major impact on the occurrence and development of AKI. This review systematically analyzes existing evidence, which suggests that the senescence of PTECs may have a dual effect. Acute cellular senescence typically mitigates uncontrolled replication of damaged cells by inducing cell cycle arrest, thereby limiting the further expansion of tissue damage. In contrast, the pathological retention of chronic senescent cells and the excessive production of the senescence-associated secretory phenotype (SASP) exacerbate the local inflammatory response and the process of fibrosis, accelerating the transformation of AKI into CKD. Despite incomplete elucidation of the spatiotemporal mechanisms governing the transition from acute to chronic cellular senescence, therapeutic interventions can be precisely targeted to specific disease stages based on their characteristic progression dynamics. This review summarizes the intervention strategies applicable at different stages of AKI, including prevention, early induction of senescence, senoreverse, senolysis, and senomorphics. Additionally, we highlight potential therapeutic targets to provide a theoretical basis for optimizing clinical management.

## 1. Introduction

Acute kidney injury (AKI), a life-threatening clinical syndrome, is defined by abrupt impairment of glomerular filtration function, typically progressing within 7 days post-initial renal insult [1]. As a major global health burden, AKI affects 10–15% of hospitalized patients worldwide [2] and 50–60% of patients in the intensive care unit (ICU) [3,4,5]. The challenge is particularly severe in low- and middle-income countries due to endemic infections, water contamination, and limited medical resources [6]. Although AKI was historically viewed as a transient and self-limiting disease, emerging evidence underscores that acute renal functional deterioration correlates with long-term consequences and can affect organs other than the kidneys [7]. This impact spanning the acute and chronic phases causes AKI to result in approximately 2 million deaths worldwide each year, taking a persistent toll on both individual health and the social healthcare system [8]. Despite recent advances in research, the current management of AKI remains primarily supportive care, with no specific therapies available. In-depth analysis of the pathogenesis of AKI is of great clinical significance for identifying effective therapeutic targets and improving the prognosis of AKI patients.

**Figure 1:**
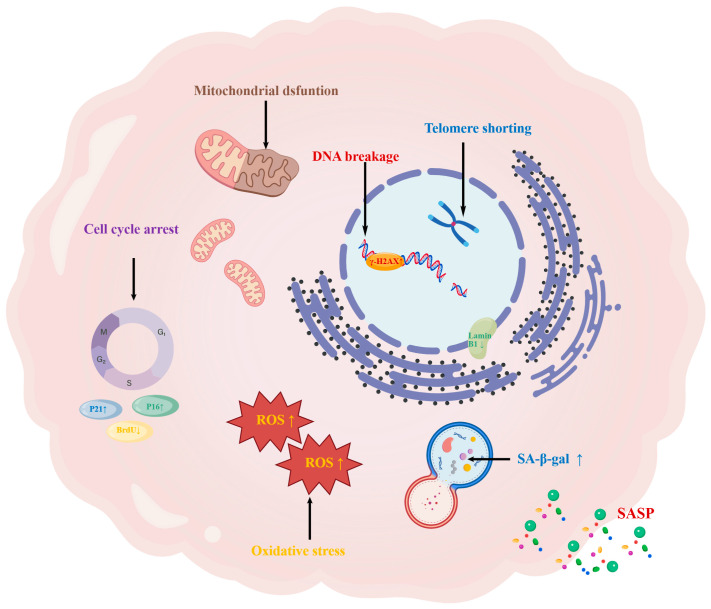
Cellular senescence-associated features. Cellular senescence is a state of irreversible cell cycle arrest induced by factors such as DNA damage, oxidative stress, mitochondrial dysfunction, telomere shortening. Senescent cells are characterized by enlarged size and irregular morphology. During the process of senescence, phosphorylation levels of γ-H2AX increase following DNA damage, while the expression of Lamin B1 is downregulated. Additionally, an increased accumulation of SA-β-gal within lysosomes can be observed, accompanied by the manifestation of the SASP phenotype. Created with BioGDP.com [16].

The study of cellular senescence, a key biological phenomenon, can be traced back to the 1961 discovery of human diploid cell strains by Leonard Hayflick and Paul S. Moorhead [9]. With advancing research, it has become clear that this phenomenon is primarily induced by stress stimuli or specific physiological processes, such as DNA damage, oxidative stress, mitochondrial dysfunction, telomere shortening, and repeated cell division [10,11,12,13]. During the process of cellular senescence, the phosphorylation levels of γ-H2AX increase following DNA damage, the expression of Lamin B1 is downregulated, and an increase in the content of SA-β-gal within lysosomes can be observed. Its core characteristics include macromolecular damage, persistent and irreversible cell cycle arrest, metabolic reprogramming, and the formation of the senescence-associated secretory phenotype (SASP). At the morphological level, senescent cells exhibit distinct characteristics, such as increased size and a flattened shape [14,15] (Figure 1).

Today, it is appreciated that the process exerts a twofold influence on the organism. It supports early development, post-injury healing, structural renewal and the restraint of cancer initiation. Yet, when defective or lingering senescent cells build up, they foster the emergence of degenerative disorders and the progressive decline of tissues with age [17]. In the kidney, these general aging mechanisms are intensified by the organ’s unique physiological architecture. Emerging evidence identifies cellular senescence as a critical mediator in the pathophysiology of AKI [18,19]. During AKI progression, multiple renal cell types, including podocytes, renal tubular epithelial cells (RTECs), vascular smooth muscle cells (VSMCs), and endothelial cells, undergo senescence. However, proximal tubular epithelial cells (PTECs) are particularly vulnerable [12,20]. As the principal functional units of the kidney, PTECs reabsorb nearly 100% of filtered glucose, albumin, phosphates, amino acids, and other organic solutes, alongside 65% to 80% of water and sodium, transporting them back into the systemic circulation via the extracellular matrix (ECM). This immense transmembrane transport burden renders PTECs highly sensitive to microenvironmental stress. Under conditions of ischemia, hypoxia, or external insult, PTECs are subjected to dual toxicity from circulating nephrotoxins and highly concentrated intraluminal metabolic wastes. Such pathological stimuli disrupt the mitochondrial electron transport chain (ETC) within PTECs, initiating oxidative stress [21]. The subsequent accumulation of reactive oxygen species (ROS) aggravates mitochondrial dysfunction while mediating DNA damage [22] and telomere attrition [23]. These processes ultimately drive PTECs to become the most senescence-susceptible cell population within the kidney [24]. In AKI, cellular senescence of PTECs has a dual impact. While cellular senescence promotes cellular repair and enhances injury resistance, persistent cell cycle arrest in PTECs exacerbates renal damage and worsens AKI prognosis [25,26]. Therefore, this review summarizes the role of cellular senescence in different stages of AKI. By integrating the latest advances in anti-aging research, it explores the potential for targeted therapeutic interventions during distinct phases of AKI.

## 2. Acute Cellular Senescence

Based on their induction and function, senescent cells are classified as either acute or chronic (Table 1). Acute cellular senescence represents a beneficial physiological mechanism. Stimuli target specific cell populations, triggering a process that is self-organized and eventually eliminated via SASP-mediated immune recruitment. This pathway is defined by its precision, transient nature, and rapid, highly regulated clearance [27].

Different organs exhibit variations in the occurrence and regulation of acute cellular senescence, although their fundamental mechanisms share many similarities. These commonalities and unique characteristics provide opportunities for cross-disciplinary integration, including insights into how the mechanisms of acute cellular senescence can be leveraged to improve renal repair. During embryonic development, senescent cells are activated at specific stages and locations to fulfill developmental functions, after which they are eliminated via programmed apoptosis or macrophage-mediated clearance [28]. This mechanism is conserved in the salamander limb regeneration model, where senescent cells emerge at defined intervals to secrete the SASP. Their subsequent rapid clearance by macrophages prevents cellular accumulation and maintains the tissue’s regenerative capacity [29]. In the context of oncogenesis, acute senescence functions as a cell-cycle arrest mechanism triggered by DNA damage or oncogenic signaling. The resulting SASP recruits immune cells to the lesion site to facilitate the elimination of potentially malignant cells [30,31]. Research on tissue repair further indicates that senescent fibroblasts and endothelial cells promote wound healing through the secretion of platelet-derived growth factor AA (PDGF-AA) [32]. Similarly, in liver injury models, the activation of the senescence program in hepatic stellate cells facilitates their recognition and clearance by natural killer (NK) cells, a process associated with the reversal of liver fibrosis [33]. Given the conserved role of acute cellular senescence in these physiological and pathological processes, it is plausible that similar regulatory mechanisms govern renal injury and repair. Damaged PTECs may trigger a transient senescence program to initiate reparative signaling, followed by immune-mediated clearance to facilitate tissue reconstruction. Consequently, elucidating the initiation, function, and resolution of acute senescence in the kidney will not only deepen the understanding of renal regenerative biology but also identify novel therapeutic targets for mitigating fibrosis and promoting functional recovery.

## 3. Chronic Cellular Senescence

Unlike acute cellular senescence, chronic cellular senescence occurs when cells are exposed to different types of stress over an extended period or experience macromolecular damage. During this process, the sluggish cell cycle transforms into a stable and irreversible cell-cycle arrest. Additionally, as the immune system deteriorates with age, the efficient clearance of senescent cells becomes impaired [34,35]. A growing number of senescent cells amass persistently and release the SASP via autocrine or paracrine pathways. The SASP varies by cell type, tissue, genetics, and trigger, and dynamically evolves over time, increasing its complexity [36]. Although SASP is initially beneficial for tissue repair, normal tissue development, and the recruitment of immune cells, its persistent presence might give rise to enduring inflammatory reactions, induce more severe cellular senescence, and ultimately lead to organ dysfunction [12,27,37,38].

Mechanistically, this process is implicated in diverse maladies associated with the aging process. In conditions such as atherosclerosis [39], neurodegenerative diseases [40,41], type 1 diabetes [42], and cartilage degradation [43], chronic cellular senescence characterized by the sustained release of the SASP has emerged as a central pathological hub linking the aging process to multisystem degenerative disorders. Chronic cellular senescence, through the sustained release of SASP, has become a central pathological hub linking aging to multisystem degenerative disorders. Targeted clearance of senescent cells or modulation of their microenvironment is now providing transformative therapeutic paradigms for age-related diseases. In AKI, chronic cellular senescence is a pivotal driver of impaired injury repair and the subsequent progression to CKD. Persistent cell-cycle arrest following the initial insult forces PTECs, endothelial cells, and interstitial cells into an irreversible senescent state. Driven by the sustained release of the SASP, this process triggers localized chronic inflammation, heightened oxidative stress, and aberrant extracellular matrix deposition [12,44,45]. Consequently, the targeted clearance of senescent cells or the modulation of the SASP has emerged as a promising therapeutic strategy to halt chronic progression and improve long-term renal outcomes.

## 4. Acute and Chronic Cellular Senescence of PTECs in AKI

It is estimated that after AKI, approximately 70% of the surviving and quiescent PTECs can re-enter the cell cycle within the initial 24 h, promoting the recovery of renal function through regenerative mechanisms [46,47]. However, some cells cannot be completely repaired and will undergo cellular senescence [48]. Multiple studies have confirmed that cellular senescence can shift the repair process toward fibrosis. For example, the removal of senescent PTECs through genetic ablation has been shown to reduce renal fibrosis [49]. Similarly, in mouse models of unilateral renal IRI and multiple administrations of cisplatin, senolytic drugs can relieve renal fibrosis [50]. Nevertheless, cellular senescence also exerts protective effects during the early phase of AKI. Employing small-molecule reversible inhibitors of cyclin-dependent kinases 4/6 (CDK4/6) during the early phase of AKI can trigger a temporary halt in the cell cycle of PTECs. This, in turn, helps evade DNA damage and mitigates renal injury [51]. This dual role also appears to be mechanistically distinct. At the molecular level, the p53/p21 signaling pathway serves as a core regulatory axis of cellular senescence. In response to stimuli such as DNA damage, metabolic stress, or ischemia–reperfusion injury (IRI), p53 is activated and subsequently upregulates the expression of its downstream target gene, p21, thereby inducing G1/S phase cell cycle arrest [52,53]. In a cisplatin-induced AKI model, mice with p21 gene deletion exhibit more severe kidney injury, indicating that p21-mediated cell cycle arrest plays a protective role during the acute phase [50]. Conversely, the p16INK4a/Rb pathway may be more central to “chronic senescence.” In the INK-ATTAC transgenic mouse model, the early clearance of cells with overexpression of p16Ink4a does not improve renal function. Instead, the clearance of senescent cells one week after the injury is beneficial for the recovery of renal function [54]. Furthermore, the knockdown of p16 not only inhibits PTEC senescence but also attenuates its paracrine proliferative effects on fibroblasts, thereby alleviating renal fibrosis [55]. These studies indicate that senescent PTECs play a dual role after AKI and seem to exhibit a temporal pattern. A recent study showed that acute senescent cells produced within 3 days after AKI will be cleared within 15 days, while chronic senescent cells produced during the chronic phase of AKI recovery will persist [24]. Based on the aforementioned research evidence, it can be inferred that acute senescent cells that emerge following AKI may exert beneficial effects during the early stages of damage, whereas chronic senescent cells that persist over time are more likely to drive the progression of fibrosis. However, the mechanisms underlying the generation of acute and chronic cellular senescence in PTECs have not yet been fully elucidated.

A study using an inducible senescence mouse model extensively investigated the heterogeneity and characteristics of primary and secondary cellular senescence in the liver and colon. The findings demonstrated that senescent cells can induce secondary senescence in neighboring cells through the secretion of SASP factors via a paracrine mechanism. Furthermore, the phenotype of secondary senescence is co-regulated by two key factors: the composition and activity of SASP factors secreted by primarily senescent cells, and the intrinsic properties of the recipient surrounding cells. Mechanistically, the study identified macrophage-derived interleukin-1β (IL-1β) as a critical signaling molecule mediating the induction of secondary senescence [56]. While another study on SASP effects demonstrates that: brief exposure to SASP can induce cellular plasticity, enhance the expression of stem cell markers, and promote tissue regeneration; whereas prolonged exposure induces intrinsic cellular senescence and proliferation arrest, thereby playing a role in suppressing abnormal regeneration [57]. Although direct evidence characterizing the dual roles of SASP specifically within the renal microenvironment remains limited, cross-organ evidence highlights the context- and time-dependent nature of key SASP components, offering mechanistic insights into the acute-to-chronic senescence transition in PTECs following AKI (Table 2).

**Figure 2:**
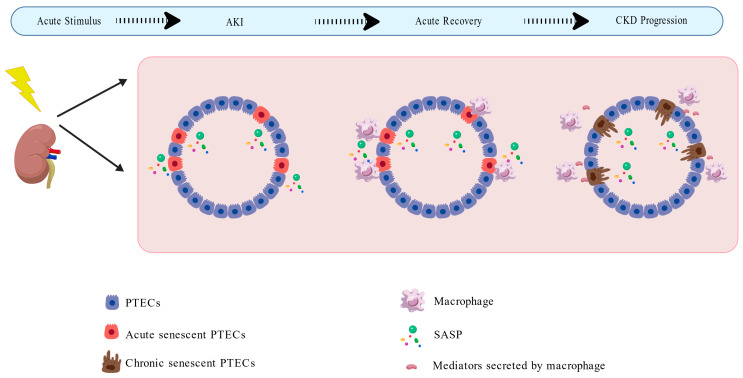
The possible mechanisms of the sequential occurrence of acute and chronic cell senescence. Based on the aforementioned research, we propose the following hypothesis: AKI is a pathological process triggered by acute stimuli in the body (indicated by the lightning symbol), which can induce the generation of acutely senescent PTECs. These cells secrete SASP factors, which promote the regeneration and repair of neighboring cells while simultaneously recruiting immune cells (represented by macrophages in the schematic) to clear the senescent PTECs. However, sustained SASP signaling, combined with mediators secreted by immune cells, may drive the formation of chronic senescent PTECs. Such cells potentially evade immune surveillance and clearance by expressing distinct signaling molecules, thereby persisting in tissues over the long term and promoting maladaptive repair, which eventually leads to CKD progression and fibrosis. Abbreviations: AKI, acute kidney injury; CKD, chronic kidney disease; PTECs, proximal tubular epithelial cells; SASP, senescence-associated secretory phenotype. Created with BioGDP.com [16].

Based on the aforementioned research, we propose the following hypothesis: AKI is a pathological process triggered by acute stimuli in the body, which can induce the generation of acutely senescent cells. These cells secrete SASP factors, which promote the regeneration and repair of neighboring cells while simultaneously recruiting immune cells to clear the senescent cells. However, sustained SASP signaling, combined with mediators secreted by immune cells, may drive the formation of chronic senescent cells. Such cells potentially evade immune surveillance and clearance by expressing distinct signaling molecules, thereby persisting in tissues over the long term and promoting chronic pathological processes such as fibrosis (Figure 2). Recently, a study on the functional heterogeneity of aged cells in the liver constructed an animal model capable of real-time tracing the dynamic evolution of aged cells and developed a comprehensive genetic tool system, enabling the direct observation of the spatiotemporal dynamics of aged cells during physiological and pathological processes [65]. This provides important methodological support for verifying the aforementioned hypothesis. If this hypothesis is confirmed, it would mean that differentiated strategies need to be adopted for interventions at different stages after kidney injury: in the early stage of injury, the aging-related pro-regenerative signaling pathways can be moderately enhanced to support tissue repair; while in the later stage, targeted and clearance of chronic aged cells that have acquired immune escape capabilities should be performed. This “staged intervention” concept holds promise for providing new insights into the precise treatment of clinical kidney injury.

## 5. Stage-Specific Therapeutic Approaches

**Figure 3:**
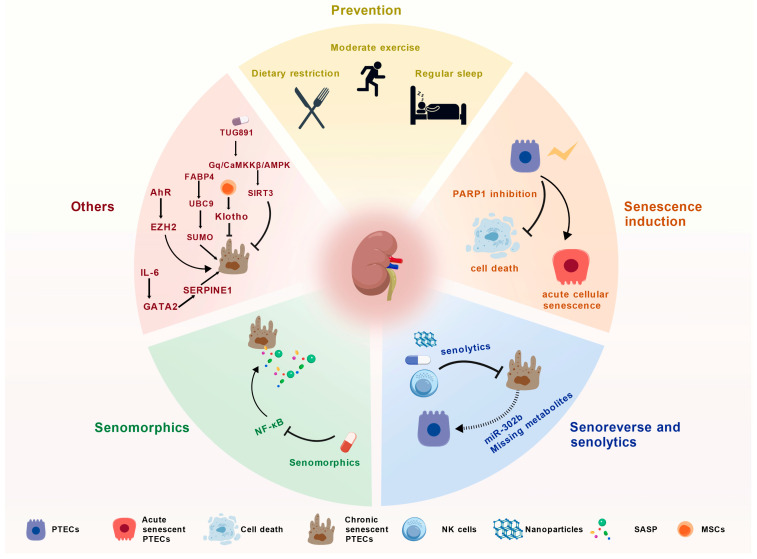
Therapeutic strategies and potential molecular targets for PTEC senescence in AKI. This schematic systematically illustrates five major categories of therapeutic strategies and their potential molecular targets addressing PTEC senescence during the progression of AKI. (1) Prevention: Emphasizes lifestyle interventions to enhance renal resilience, including dietary restriction, moderate exercise, and regular sleep, aiming to delay cellular senescence through systemic metabolic regulation. (2) Senescence induction: Focuses on precise intervention during the early stages of injury. By utilizing PARP1 inhibition to reduce injury-induced PTEC cell death, this strategy redirects cells toward a controlled, temporarily protective state of acute cellular senescence to facilitate tissue repair. (3) Senoreverse and senolytics: Senoreverse strategies attempt to revert chronic senescent PTECs back into functional cells using miR-302b or by supplementing missing metabolites. Senolytic strategies focus on specifically recognizing and eliminating irreversible chronic senescent PTECs via pharmacological agents, nanoparticle delivery systems, or NK cells. (4) Senomorphics: Centers on microenvironmental regulation by inhibiting the NF-κB pathway to suppress the release of the SASP, thereby mitigating the inflammatory storm and preventing the propagation of senescence signals to neighboring cells. (5) Others: Encompasses complex, AKI-specific regulatory networks, including SIRT3 signaling, the FABP4–UBC9-mediated SUMOylation process, the AhR–EZH2 and IL-6–GATA2 axes, and the paracrine protective effects exerted by MSCs via Klotho secretion. The lightning symbol represents acute stimulation; Solid arrows indicate promoting or inducing effects; solid bars represent inhibitory or blocking effects; dashed lines denote indirect regulatory relationships or those yet to be fully validated. Abbreviations: AhR, aryl hydrocarbon receptor; AKI, acute kidney injury; AMPK, AMP-activated protein kinase; CaMKKβ, calcium/calmodulin-dependent protein kinase kinase beta; EZH2, enhancer of zeste homolog 2; FABP4, fatty acid-binding protein 4; GATA2, GATA binding protein 2; Gq, G protein subunit alpha q; IL-6, interleukin 6; MSCs, mesenchymal stem cells; NF-κB, nuclear factor kappa B; NK cells, natural killer cells; PARP1, poly(ADP-ribose) polymerase 1; PTECs, proximal tubular epithelial cells; SASP, senescence-associated secretory phenotype; SERPINE1, serpin family E member 1; SIRT3, sirtuin 3; SUMO, small ubiquitin-like modifier; UBC9, ubiquitin carrier protein 9. Created with BioGDP.com [16].

Although the specific mechanisms have not yet been fully elucidated, based on current research progress, we can summarize stage-specific intervention strategies that may be applicable to individuals with AKI. Specifically, early preventive measures can be implemented for populations at risk of AKI. During the regenerative repair phase, the generation of acute senescent cells can be moderately promoted to support tissue repair. In the chronic phase, it is essential to promptly reverse or eliminate chronic senescent cells and neutralize their secreted SASP factors (Figure 3).

### 5.1. Preventing AKI

#### 5.1.1. Dietary Restriction

Osborne first discovered that dietary restriction (DR) without causing malnutrition can maximize the lifespan of rodents and improve their health status. Subsequent studies have further confirmed the efficacy of DR in disease prevention and life extension. In humans, DR can promote adaptive changes, thereby preventing various pathological changes, including renal fibrosis [66,67]. As a form of DR, how calorie restriction (CR) affects cellular senescence has become a hot topic of research. Through fasting and reducing calorie intake, CR delays various hallmarks of cellular senescence. Reducing the intake of dietary protein, carbohydrates, and fat all play a role in regulating senescent cells in multiple tissues and cell types. However, there are still many unknowns regarding how these diets affect cellular senescence through their physiological and molecular mechanisms [68]. Alireza Raji-Amirhasani et al. studied the effects of four dietary patterns—CR, intermittent fasting (IF), time-restriction eating (TR), and high-fat diet (HF). The research findings indicate that TR and CR can exert renal protective effects by downregulating TGF-β1 levels and reducing malondialdehyde (MDA) content following AKI, while simultaneously upregulating SIRT1 expression, enhancing total antioxidant capacity, and improving creatinine clearance. These findings suggest that CR and TR may represent potential preventive strategies for AKI, warranting further investigation in clinical settings [69]. Additionally, CR can improve renal dysfunction, oxidative stress, apoptosis, and inflammatory responses induced by IRI by modulating gut microbiota *P. goldsteinii* and the serum metabolite dodecafluorpentan and at the same time enhance the intestinal barrier function [70]. A recent study further demonstrates that IF can alleviate renal fibrosis following IRI by suppressing cellular senescence [71]. These findings collectively highlight the significant potential of DR in ameliorating AKI.

#### 5.1.2. Regular Sleep and Moderate Exercise

Multiple studies have indicated that sleep is indispensable in physiological regulation when it comes to maintaining the health of animal models and humans. However, a decline in sleep quality, especially sleep disorders such as sleep fragmentation, sleep deprivation, and insomnia, may have a systemic impact on the body’s health through multiple mechanisms. Research indicates that sleep deprivation can promote the production of the SASP by upregulating the expression of DNA damage response genes, thereby accelerating the process of cellular senescence [72]. Notably, for every 1-point increase in the healthy sleep score, the hazard ratio for incident acute kidney injury is 0.95 (95% CI, 0.93–0.97), which indicates that the higher the healthy sleep score (meaning a healthier sleep pattern), the lower the risk of incident AKI [73]. At the same time, moderate exercise has also been proven to be effective in delaying cellular senescence and can prevent the occurrence of AKI by improving the body’s metabolic state [74,75,76]. Long-term regular moderate exercise can significantly reduce cellular senescence and inflammation levels while enhancing betaine metabolism. Exercise-induced betaine enrichment (partially mediated by renal biosynthesis) exerts anti-aging protective effects. However, it should be expressly pointed out that excessive and strenuous exercise may lead to rhabdomyolysis, and this secondary damage will instead increase the risk of AKI [77]. Furthermore, betaine supplementation has been demonstrated to mimic the effects of exercise and represents a viable nutritional intervention strategy for delaying aging [78]. Therefore, establishing a regular sleep–wake rhythm combined with moderate exercise and supplemented with betaine may serve as an effective intervention strategy to synergistically prevent cellular senescence and reduce the risk of AKI.

### 5.2. Conversion of Cell Death into Senescence in the Early Stage of AKI

Poly (ADP-ribose) polymerase 1 (PARP1) is a ribozyme widely expressed within cells and plays an important role in various cellular processes such as DNA damage repair, apoptosis, and gene transcription regulation [79]. Currently, PARP inhibitors have been developed for cancer treatment and have shown relatively good therapeutic effects, especially in tumors such as breast cancer and ovarian cancer with BRCA gene mutations [80]. A recent study has shown that in the model of unilateral renal ischemia–reperfusion injury (uIRI), PARP inhibition reduces PTECs necrosis and increases acute senescence. It not only ameliorates the degree of tissue damage but also effectively inhibits the progression of long-term renal fibrosis. It is worth noting that this inhibitory strategy does not lead to the continuous accumulation of chronic senescent cells. These data suggest that converting cell death into acute cellular senescence may play an important role in the early treatment of AKI [81]. Additional molecular candidates have been identified with potential relevance to early-stage AKI intervention, though direct evidence in AKI models remains limited. Minichromosome maintenance 4 (MCM4), a regulator of cell proliferation and cell cycle progression, was found to be upregulated in PTECs following AKI, with expression peaking during the acute phase and declining at day 4—a time window coinciding with acute senescence. Whether enhancing MCM4 expression confers protective effects requires further investigation [82]. Similarly, activation of the purinergic receptor P2Y purinoceptor 2(P2RY2) by the approved agonist diquafosol promoted tubular repair in a zebrafish pronephros model [83]; its efficacy in mammalian AKI models remains to be established.

### 5.3. Senolytics or Functional Restoration of Senescent Cells

#### 5.3.1. Senoreverse Reverses Cellular Senescence

In previous studies, due to the fact that chronic cellular senescence leads to an irreversible arrest of the cell cycle, researchers had never explored whether it was possible to reverse cellular senescence and restore its proliferative capacity. Recently, a study has scientifically demonstrated the possibility that cellular senescence can be reversed and proposed an innovative “Senoreverse” strategy for reversing cellular senescence [84]. Human embryonic stem cell-derived exosomes (hESC-Exos) are rich in various proteins and microRNAs (miRNAs), and have the potential to regulate senescence and proliferation [85]. Based on this, researchers treated high-passage senescent fibroblasts with hESC-Exos. They observed a significant decrease in multiple senescence markers, accompanied by an increase in proliferation-related indicators. These findings indicate that hESC-Exos can effectively reverse the senescent phenotype. In addition, in the natural aging mouse model, hESC-Exos also demonstrated significant anti-aging effects. It can not only reshape the composition of cell subsets in the liver and skin but also restore the proliferative capacity of senescent cells. Through in-depth analysis of the proteins and miRNAs in hESC-Exos, the research team discovered a miRNA named miR-302b, which may be a key factor in reversing senescence. More importantly, miR-302b has good safety and can delay aging while avoiding obvious safety hazards [84]. Researchers have integrated single-cell live imaging and mass spectrometry (SCLIMS) to simultaneously capture metabolomic information and phenotypic characteristics at the single-cell level, establishing correlation analysis between the two. This study revealed the close relationship between cellular metabolic heterogeneity and the aging process. Particularly noteworthy is that targeted supplementation of core metabolites missing in senescent cells (such as taurine and phosphocreatine) can restore the cellular metabolome to a normal state, significantly extending the lifespan of Caenorhabditis elegans by 33–50% without observable side effects [86].

These findings introduce the concept of “senoreverse” as a potential therapeutic paradigm. However, it is important to note that neither hESC-Exos/miR-302b nor metabolite supplementation has been evaluated in AKI models to date. Whether these strategies can reverse PTEC senescence or improve outcomes following AKI remains an open question requiring dedicated investigation.

#### 5.3.2. Senolytics Therapy Selectively Eliminates Senescent Cells

Senolytics therapy is a treatment strategy targeting senescent cells, aiming to selectively eliminate these cells to delay or reverse aging-related diseases and pathological processes. At present, senolytics have been proven to alleviate chronic diseases in pre-clinical animal models, such as pulmonary fibrosis [87], vascular calcification [88], Alzheimer’s disease [89], obesity [90], and hepatic steatosis [91]. In addition, there are also some studies that have investigated the mechanisms and effects of senolytics therapy in AKI. The B-cell lymphoma 2(BCL-2) protein family, including BCL-2, BCL-W, BCL-XL, MCL-1 and A1, assumes a crucial function in controlling cell death via diverse means. Among them, BCL-W and BCL-XL are responsible for the majority of the resistance of senescent cells to apoptosis. Early studies have shown that mice treated with ABT-737, an inhibitor targeting BCL-W and BCL-XL, could effectively clear the senescent cells induced by DNA damage in the lungs. Moreover, it was also capable of eliminating the senescent cells that formed in the epidermis due to the activation of P53 by the transgenic p14ARF [92]. Emerging evidence reveals that pharmacological inhibition of BCL2/w/xl via ABT-263 effectively mitigates cellular senescence in renal tissues, thereby reinstating regenerative potential. Experimental investigations demonstrate that pretreatment with this agent prior to ischemia–reperfusion injury confers multifaceted renal protection, including enhanced proliferative capacity of tubular epithelial cells, improved functional recovery of renal filtration, and attenuated progression of fibrotic pathology [93]. Forkhead box protein O4 (FOXO4) plays a crucial role in the regulation of cellular senescence through its specific interaction with the p53 protein. Research indicates that the formation of the FOXO4-p53 complex can significantly upregulate the transcriptional level of the senescence-associated protein p21, thereby inducing the process of cellular senescence. Based on this molecular mechanism, researchers have developed a peptide inhibitor named FOXO4-DRI, which can specifically interfere with the interaction between FOXO4 and p53. In senescent cells, FOXO4-DRI can induce the nuclear translocation of p53, thus initiating the endogenous apoptosis program of the cells [94]. In a study of folic acid (FA)-induced renal injury, FOXO4-DRI successfully cleared senescent cells. However, it failed to significantly ameliorate renal fibrosis progression and structural damage. This limitation is likely constrained by specific microenvironmental factors or the therapeutic time window [49].

Quercetin, a naturally occurring flavonoid compound, can be used either alone or in combination with Dasatinib, representing one of the most extensively studied senolytic strategies to date. Research has demonstrated that the combined administration of Quercetin and Dasatinib specifically induces apoptosis in PTECs and significantly ameliorates the degree of renal fibrosis in a mouse model of uIRI [49].

Additional strategies have shown senolytic potential in non-AKI contexts or remain at the mechanistic hypothesis stage: Some researchers have found that after IRI occurs, DcR2 accelerates the senescence process of RTECs, thereby promoting the progression of renal fibrosis [95]. In diabetic nephropathy (DN), DcR2 mediates the apoptosis-resistant phenotype of senescent RTECs by enhancing the interaction between GRP78 and caspase 7 and promoting the phosphorylation of Akt [96]. Although these studies focus on the broader population of RTECs, their findings provide important context for understanding the highly susceptible PTECs subset. Whether DcR2 inhibition can selectively eliminate senescent PTECs in AKI warrants further investigation.

Recent advances in biomaterials have provided new strategies for targeting senescent cells. For example, researchers have developed a senolytic platform that selectively eliminates senescent cells. This platform innovatively uses lipofuscin accumulated in senescent cells as a specific target and precisely delivers the senolytic drug dasatinib to senescent cells via nanocarriers, thereby significantly reducing toxicity to normal cells [97]. Additionally, other research teams have exploited the unique metabolic and iron homeostasis abnormalities in senescent cells to construct self-illuminating nanoplatforms. These platforms spatiotemporally couple photodynamic effects with ferritin-mediated ferroptosis, enabling precise elimination of senescent cells without requiring external light irradiation [98].

Furthermore, studies have shown that cationic mesoscale (350~400 nm) can selectively accumulate in PTECs [99,100]. However, whether they can specifically bind to and eliminate senescent PTECs remains to be further explored. These advancements offer new potential strategies for intervening in disease progression following AKI.

The SASP is a crucial mediator of the effects of senescent cells. It activates immune surveillance mechanisms through the secretion of factors, eliminates senescent cells, and promotes the restoration of tissue homeostasis [33]. Senescent cells’ clearance is significantly associated with the action of NK cells [101]. Current studies have revealed that treatment with DPP-4 antibodies promotes the preferential elimination of senescent fibroblasts by NK cells [102], while targeting SMARCA4 enhances NK-mediated clearance of senescent cells [103]. These studies suggest that enhancing the senescent cell-killing activity of NK cells represents a feasible intervention strategy. Further, in-depth research can be conducted to explore its application in AKI.

### 5.4. Senomorphics Block the Aging Pathway and SASP Production

The principle of senomorphics is to restore the function of senescent cells and inhibit the SASP, thereby reducing inflammation and functional impairment. Notably, nuclear factor κB (NF-κB), as a key regulatory hub of SASP, activates the transcriptional network of inflammatory factors such as IL-6 and TNF-α through its dynamic nuclear translocation, playing a central role in the formation of the senescent microenvironment [104]. Beyond direct transcriptional activation, the interaction between NF-κB and histone deacetylases (HDACs) significantly influences the early inflammatory environment in damaged tubules. Recent evidence suggests that these epigenetic modulators refine the regulatory strategies for senescent cell phenotypes, where HDAC-mediated deacetylation of NF-κB subunits can either sustain or mitigate the pro-inflammatory response depending on the injury context [105]. Therefore, targeting NF-κB and its associated epigenetic machinery holds promise as an intervention to modulate SASP and alleviate senescence-related inflammatory injury.

Rapamycin can interfere with the IL-1A-NF-κB feedback loop by inhibiting the mammalian target of rapamycin (mTOR), thereby suppressing the secretion of selected SASP components [106]. In AKI, the role of rapamycin shows certain contradictions. Research indicates that, on the one hand, rapamycin may alleviate the inflammatory response and cell apoptosis by inhibiting the mTORC1 signalling pathway, thus protecting the kidneys [106,107]. For instance, in LPS-induced AKI mouse models, rapamycin treatment significantly lowers creatinine levels, preserves renal parenchyma, and demonstrates anti-aging effects [108]. On the other hand, its ability to inhibit cell proliferation and repair may delay the regeneration of PTECs and exacerbate kidney injury. For example, in models of IRI, its protective effects are inconsistent [107,109,110]. This discrepancy may be closely related to factors such as the stage of injury, drug dosage, and individual differences. Therefore, further research is still required to clarify its mechanisms of action and optimize corresponding treatment strategies.

Metformin [111] can effectively inhibit the production of the SASP by suppressing the activation of NF-κB in a variety of pathological conditions, including cancer inhibition [112], vascular protection [113], and fibrosis prevention [114]. However, in AKI models, metformin exhibits unexpected potential for nephrotoxicity. This phenomenon is related to the expansion of neutrophils and the induction of NETosis [115]. Thus, despite its well-established senomorphic effects in other disease contexts, metformin cannot be directly extrapolated to AKI without further evaluation. These findings underscore the critical importance of disease-specific pharmacological evaluation.

As one of the most essential mediators for inflammation resolution, Lipoxin A4 (LXA4) activates anti-inflammatory pathways precisely at the physiological end of the acute inflammatory phase [116]. LXA4 can inhibit the NF-κB-mediated inflammatory response and the p53/p21 senescence pathway both in vivo and in vitro. In addition, in the rat model of AKI induced by sepsis, pretreatment with LXA4 can inhibit renal inflammation and the senescence of PTECs after AKI. Ultimately, it can significantly restore the renal function of rats and improve their survival rate [117].

Numerous studies have demonstrated that various natural flavonoids, such as hypericin [118], rutin [119,120], and resveratrol [121] can exert important renal protective effects during the pathological progression of AKI by modulating NF-κB-mediated inflammatory responses. These compounds are expected to become safer options.

Ginkgo biloba biflavone (Ginkgetin) is a key component in Ginkgo biloba extract responsible for its anti-aging and anti-inflammatory effects. Researchers have discovered that Ginkgetin can directly bind to the STING protein, thereby inhibiting the activation of the cGAS-STING signaling pathway. This, in turn, reduces the activation of downstream transcription factors IRF3 and NF-κB. In various senescent cell models induced by doxorubicin or ionizing radiation, Ginkgetin effectively lowers the expression levels of senescence markers (such as p16, p21, and SA-β-gal) and SASP factors (such as IL-6 and IL-1β) [122]. In sepsis-associated acute kidney injury (SA-AKI), the abnormal activation of the STING-PERK signaling pathway exacerbates the senescence and apoptosis of PTECs, thereby aggravating renal tissue damage [123]. Based on the above mechanisms, Ginkgetin, which targets the inhibition of the STING pathway, may serve as a potential therapeutic strategy for ameliorating the pathological progression of SA-AKI.

In the management of AKI, the senomorphic approach brings both opportunities and challenges. Although mTOR inhibitors and Metformin show great potential in terms of their mechanisms of action, due to the context-dependent nature of their effects, careful risk-benefit assessment is required when applying them clinically. LXA4, natural flavonoids, and Ginkgetin as highly attractive candidates, are worthy of further development. However, comparative efficacy studies and clinical validation are still essential. Future research should prioritize time-specific interventions, personalized dosing strategies, and combination therapy regimens to optimize therapeutic outcomes and minimize adverse reactions.

### 5.5. Other Cellular Senescence Targets with Application Prospects in AKI

In addition to the aforementioned general anti-aging therapies, the specific pathways that mediate cellular senescence following AKI also hold great potential as therapeutic targets. Preclinical studies have confirmed that, with the exception of one subtype, other members of the Sirtuin (SIRT) protein family play key mediating roles in the occurrence and progression of AKI [124]. Recent research reports that TUG891, an agonist of free fatty acid receptor 4 (FFAR4)—a member of the G protein-coupled receptor (GPCR) family—can upregulate SIRT3 expression through the G protein subunit alpha q (Gq) subunit-mediated Calcium/calmodulin-dependent protein kinase kinase beta (CaMKKβ)/AMP-activated protein kinase (AMPK) signaling pathway, thereby alleviating the senescence of PTECs [125]. Meanwhile, substantial evidence indicates that the SIRT family plays an important regulatory role in cellular aging mechanisms. Studies based on various models, including human vascular endothelial cells [126], mouse embryonic fibroblasts [127], macrophages under oxidative stress [128], and lung epithelial cells [129], have shown that the expression of specific SIRT subtypes (particularly SIRT1 and SIRT6) is significantly downregulated, while SIRT2 expression remains relatively stable. These findings not only confirm the differential regulatory role of the SIRT family in cellular senescence but also suggest that other SIRT subtypes may similarly participate in the regulation of cellular senescence following AKI, providing new directions for future research in this field [130,131].

During the pathological process of AKI, there is a complex interaction between lipid metabolism disorders and cellular senescence. Fatty acid-binding protein 4 (FABP4) accelerates the senescence of PTECs and exacerbates cell damage in IRI-induced AKI by regulating the expression of ubiquitin-conjugating enzyme 9 (UBC9) and its mediated SUMOylation modification process [132].

Additionally, in a cisplatin-induced AKI model, the aryl hydrocarbon receptor (AhR) in PTECs activates the p16INK4a/p21CIP1 signaling pathway by regulating the expression and methylation modification activity of histone methyltransferase Enhancer of zeste homolog 2 (EZH2), thereby accelerating the senescence process of PTECs. This finding lays an important theoretical foundation for novel AKI treatment strategies targeting the AhR-EZH2 signaling axis [133].

A research team observed that during remote ischemic preconditioning, this intervention can reduce serum IL-6 levels and alleviate renal cell senescence in a crush injury rabbit model. Further experiments revealed that Interleukin-6 (IL-6) activates the transcription factor GATA binding protein 2 (GATA2), promotes Serpin family E member 1 (SERPINE1) expression, subsequently upregulates the p53/p21 pathway, ultimately leading to PTECs senescence and G2/M phase arrest. Inhibiting IL-6 (e.g., with tocilizumab, TCZ) can block this pathway and mitigate cellular senescence [134].

Beyond that, mesenchymal stem cell (MSC) therapy also shows broad application prospects in the treatment of AKI. Existing studies have confirmed that this therapy alleviates AKI-related pathological damage and inhibits renal cellular senescence. These protective effects are primarily achieved by upregulating Klotho protein expression in renal tissues [17,135]. However, this therapy still has significant limitations at present: on the one hand, through conventional administration methods such as intravenous injection, the retention efficiency of mesenchymal stem cells in renal tissues is extremely low; on the other hand, uremic toxins produced during the course of AKI can induce senescence of MSCs themselves, thereby impairing their therapeutic efficacy [136,137]. Therefore, there is an urgent need to further explore more optimized administration strategies or cell modification schemes to break through the aforementioned bottlenecks.

The discovery of these new targets provides new treatment ideas and strategies for the treatment of AKI. By interfering with or regulating these targets, more effective treatment methods may be developed (Table 3).

## 6. Conclusions

The dual roles of PTEC senescence in AKI pose both challenges and opportunities for therapeutic intervention. It is of great significance to select appropriate treatment strategies tailored to different pathological stages. Implementing precise prevention for high-risk groups, redirecting injury-induced cell death toward a protective acute senescence early in the disease, and adopting comprehensive treatments—such as clearing chronic senescent cells or neutralizing the SASP in later stages—offer a highly promising therapeutic roadmap.

However, applying this sequential concept in clinical practice faces a major hurdle: accurately determining the patient’s precise disease stage. The transition from beneficial acute senescence to harmful chronic senescence happens rapidly, often within a narrow window of hours to days post-injury. Currently, clinicians lack reliable tools to identify this critical turning point. To guide the optimal timing of senotherapy, future research must identify and validate accessible clinical biomarkers. These could include monitoring the dynamic changes of cell-cycle proteins (such as p21 or p16) in urine or blood, tracking circulating SASP components, or standardizing the staining of senescence markers in renal biopsies. Without these real-time clinical readouts, it will be difficult to stratify patients and safely balance the beneficial and harmful effects of senescence.

In addition to diagnostic challenges, our central hypothesis regarding the transition from acute to chronic senescence requires rigorous experimental proof. Future laboratory studies should utilize in vivo lineage tracing and kidney organoid models to track the long-term fate of acutely senescent PTECs and confirm whether they directly drive the progression of CKD. Furthermore, while many effective molecular targets have been discovered, translating them into clinical applications requires further optimization of their specificity, efficacy, and safety. Future clinical trials should adopt adaptive designs, allowing treatments and dosages to be adjusted based on the patient’s continuous biomarker results.

By overcoming these translational barriers and leveraging the dual roles of PTEC senescence, we can pave the way for innovative, precision-timed treatments. These advanced approaches hold the potential to not only alleviate AKI but also prevent its maladaptive progression to CKD, ultimately improving the long-term prognosis of patients.

## Figures and Tables

**Table 1:** Acute and chronic cellular senescence.

Comparison Items	Acute Cellular Senescence	Chronic Cellular Senescence
Triggering factor	Acute increase in specific stress	Gradual increase in stress/damage
SASP	Explosive secretion of pro-inflammatory factors	Sustained low-grade inflammation
Clearance mechanism	Scheduled clearance by immune cells	Unscheduled clearance
Physiological/pathological effects	Tumor suppression, tissue repair, and embryonic development	Age-related conditions, tissue degradation, and body aging
Role in AKI	Play a beneficial role in the early stage	Promote AKI-CKD progression

**Table 2:** Dual roles of SASP during the transition from repair to chronic pathology.

Organ/Model	Acute Role	Chronic Role	Reference
Liver	Senescent hepatic stellate cells secrete IL-6 and CXCL2 to stimulate hepatocyte proliferation and drive liver regeneration	Uncontrolled accumulation and propagation of senescence in hepatic stellate cells adjacent to hepatocytes results in inflammation and steatosis, thereby exacerbating fibrosis	[58,59]
Keratinocytes	Transient exposure to the SASP provides regenerative signals that induce cellular plasticity and stemness, a process beneficial for tissue regeneration	Prolonged or aberrant SASP exposure subsequently triggers a cell-intrinsic senescence block that counteracts these growth-promoting signals, leading to paracrine senescence responses and diminished regenerative capacity	[57]
Skin	Senescent fibroblasts and endothelial cells secrete PDGF-AA, inducing myofibroblast differentiation and accelerating wound closure	Upon senescence, vascular endothelial cells secrete SASP factors that activate cutaneous nerves. This prompts the release of a neuropeptide called CGRP, which triggers excessive activation and degranulation of mast cells. Ultimately, this cascade leads to dermal thinning, collagen degradation, and delayed wound healing	[32,60]
Lung	In a young host, virus-induced senescence may play a beneficial role by recruiting neutrophils and other immune cells through the SASP, thereby facilitating viral clearance and tissue repair	Senescent lung fibroblasts secrete leukotrienes through the ALOX5 pathway, which act on cysteinyl leukotriene receptors to drive pulmonary fibrosis. In addition, downregulation of SIRT1 elevates the transcription level of IL-11 in senescent fibroblasts, consequently promoting a pro-fibrotic phenotype and the progression of pulmonary fibrosis.	[61,62,63]
Bone	NA	Senescent cells in the fracture callus secrete TGFβ, reducing mesenchymal progenitor cell numbers and delaying fracture healing	[64]

**Table 3:** Senescence-targeted Interventions in AKI Animal Models.

Intervention	Target	AKI Model	Senescence Markers	Renal Outcomes	Reference
**Genetic Models**					
*Myd88* KO	TLR/IL-1R	FA	Lamin B1 ↑; SA-β-Gal, p16 ↓	SCr, KIM-1, NGAL, TIS ↓	[49]
*Fabp4* KO	FABP4	UIRI	p21, p53, p16, SA-β-Gal, SASP, γ-H2AX ↓	BUN, SCr, NGAL ↓	[132]
**Pharmacological**					
D + Q	Bcl-2/xL	UIRI, Cis	Lamin B1, Ki-67 ↑; SA-β-Gal, p21/53/16, SASP ↓	BUN, Masson score, α-SMA ↓	[50]
PD 0332991	CDK4, CDK6	UIRI	BrdU+ ↓ (first 24h); BrdU+ ↑ (recover in 72h)	BUN, TIS ↓	[51]
IF	p21	UIRI	p21, SASP ↓	Masson score, TIS ↓	[71]
PJ34	PARP1	UIRI	p21, p16,γ -H2AX ↑; Ki-67 ↓	KIM-1, Profibrotic genes ↓	[81]
ABT-263	BCL-2/W/XL	UIRI	SA-β-Gal, p21/16, γ-H2AX ↓	KIM-1, BUN, SCr, TGF-β ↓	[93]
FOXO4—DRI	FOXO4-p53	FA	Lamin B1, Ki-67 ↑; SA-β-Gal ↓	Does not affect kidney damage and fibrosis	[49]
Rapamycin	mTOR	UIRI	SASP, Ki-67 ↓ (early stage)	SCr ↑ (early stage); Fibronectin, α-SMA, Col I ↓	[107]
Rapamycin	mTOR	LPS	SA-β-Gal, p21 ↓	SCr, TIS ↓	[108]
Rapamycin	mTOR	UIRI, Cis	Autophagy was not activated	No improvement was observed in BUN, SCr, NGAL, or TIS	[109]
LXA4	NF-κB	CLP	SA-β-Gal, p21/16, SASP, Ki-67 ↓	BUN, SCr, KIM-1, NGAL, Fibronectin, Col I ↓	[117]
C176	STING-PERK pathway	LPS	SA-β-Gal, p21/53, SASP ↓	BUN, SCr, Pathological damage ↓	[123]
Hyperin	NF-κB	Cis	SASP (TNF-α, IL-1β, IL-6) ↓	BUN, SCr ↓	[118]
Rutin	NF-κB	LPS	SASP (TNF-α, IL-6) ↓	BUN, SCr ↓	[120]
Resveratrol	NF-κB	Polymicrobial sepsis	SASP (TNF-α, IL-1β, IL-6) ↓	BUN↓, SCr↓ HO-1↓, KIM-1↓, NGAL ↓	[121]
TUG-891	FFAR4	Cis, UIRI, CLP	SA-β-Gal, Lamin B1, p21/53, γ-H2AX, SASP ↓	BUN, SCr, KIM-1, NGAL, TIS ↓	[125]
BAY2416964	AhR	Cis	SA-β-Gal, p21/53/16, SASP ↓	BUN, SCr, KIM-1, NGAL, TIS ↓	[133]

Note: All models refer to rodent studies unless specified. Intervention: Gene name KO indicates a genetic knockout model (italicized). Abbreviations: AKI Models: FA, folic acid; UIRI, unilateral ischemia–reperfusion injury; Cis, cisplatin; LPS, lipopolysaccharide; CLP, cecal ligation and puncture. Markers & Outcomes: SA-β-Gal, senescence-associated beta-galactosidase; SASP, senescence-associated secretory phenotype; SCr, serum creatinine; BUN, blood urea nitrogen; KIM-1, kidney injury molecule-1; NGAL, neutrophil gelatinase-associated lipocalin; TIS, tubular injury score; Col I, Collagen I. Interventions: KO, knockout; D + Q, Dasatinib plus Quercetin; IF, Isoflavone; LXA4, Lipoxin A4. Symbols: ↑, increased/upregulated; ↓, decreased/downregulated.

## Data Availability

Data sharing is not applicable to this article as no data were created or analysed in this study.

## Abbreviations

The following abbreviations are used in this manuscript:

AKI	Acute kidney injury
CKD	Chronic kidney disease
SASP	Senescence-associated secretory phenotype
ICU	Intensive Care Unit
LMICs	Low- and Middle-Income Countries
VSMCs	Vascular smooth muscle cells
RTECs	Renal tubular epithelial cells
PTECs	Proximal tubular epithelial cells
ECM	Extracellular matrix
ETC	Electron transport chain
ROS	Reactive oxygen species
PDGF-AA	platelet-derived growth factor AA
NK	Natural killer
CDK4/6	Cyclin-dependent kinases 4/6
IRI	Ischemia-reperfusion injury
IL-1β	Interleukin-1β
DR	Dietary restriction
CR	Calorie restriction
IF	Intermittent fasting
TR	Time-restriction eating
HF	High-fat diet
MDA	Malondialdehyde
PARP1	Poly (ADP-ribose) polymerase 1
uIRI	Unilateral renal ischemia–reperfusion injury
MCM4	Minichromosome maintenance 4
P2RY2	P2Y purinoceptor 2
hESC-Exos	Human embryonic stem cell-derived exosomes
SCLIMs	Single-cell live imaging and mass spectrometry
BCL-2	B-cell lymphoma 2
FOXO4	Forkhead box protein O4
FA	Folic acid
DN	Diabetic nephropathy
NF-κB	Nuclear factor κB
HDACs	Histone deacetylases
mTOR	Mechanistic target of rapamycin
LXA4	Lipoxin A4
SIRT	Sirtuin
FFAR4	Free fatty acid receptor 4
GPCR	G protein-coupled receptor
SUMO	Small ubiquitin-like modifier
Gq	G protein subunit alpha q
CaMKKβ	Calcium/calmodulin-dependent protein kinase kinase beta
AMPK	AMP-activated protein kinase
EZH2	Enhancer of zeste homolog 2
IL-6	Interleukin-6
GATA2	GATA binding protein 2
SERPINE1	Serpin family E member 1
FABP4	Fatty acid-binding protein 4
UBC9	ubiquitin-conjugating enzyme 9
AhR	Aryl hydrocarbon receptor
MSC	mesenchymal stem cell
